# Synthesis, characterization, and lead removal efficiency of orange peel powder and orange peel powder doped iron (III) oxide-hydroxide

**DOI:** 10.1038/s41598-023-38035-7

**Published:** 2023-07-04

**Authors:** Pornsawai Praipipat, Pimploy Ngamsurach, Tanyaporn Joraleeprasert

**Affiliations:** 1 Department of Environmental Science, Khon Kaen University, Khon Kaen, 40002 Thailand; 2 Environmental Applications of Recycled and Natural Materials (EARN) Laboratory, Khon Kaen University, Khon Kaen, 40002 Thailand

**Keywords:** Engineering, Materials science

## Abstract

Lead contamination in wastewater causes toxicity to aquatic life, the environment, and water quality, and it causes many human dysfunctions and diseases. Thus, it is necessary to remove lead from wastewater before discharging it into the environment. Orange peel powder (OP) and orange peel powder doped iron (III) oxide-hydroxide (OPF) were synthesized, characterized, and investigated lead removal efficiencies by batch experiments, adsorption isotherms, kinetics, and desorption experiments. The specific surface area of OP and OPF were 0.431 and 0.896 m^2^/g, and their pore sizes were 4.462 and 2.575 nm, respectively which OPF had a higher surface area than OP, whereas its pore size was smaller than OP. They were semi-crystalline structures that presented the specific cellulose peaks, and OPF also detected the specific iron (III) oxide-hydroxide peaks. The surface morphologies of OP and OPF were irregular and porous surfaces. Carbon (C), oxygen (O), calcium (Ca), O–H, C–H, C=C, C–O, C=O, and –COOH were observed in both materials. The pH_pzc_ of OP and OPF were 3.74 and 4.46. For batch experiments, OPF demonstrated a higher lead removal efficiency than OP because of spending less on material dosage than OP, and OPF demonstrated high lead removal by more than 95% while OP could remove lead at only 67%. Thus, the addition of iron (III) oxide-hydroxide helped to increase material efficiency for lead adsorption. Both materials corresponded to the Freundlich model relating to physiochemical adsorption, and they also corresponded to a pseudo-second-order kinetic model relating to a chemisorption process. Moreover, both materials could be reusable for more than 5 cycles for lead adsorption of more than 55%. Therefore, OPF was potential material to apply for lead removals in industrial applications.

## Introduction

Lead contaminations in natural water or wastewater are a concern because of its high toxicity to aquatic life, the environment, and water quality and consumption. Lead accumulations through the food chain might cause many human diseases as well such as human cancer, anemia, paralysis, and lead poisoning^1^. Many industries of battery, dye and pigment, plastic, steel, electronic, and pesticides are sources of lead discharging to the environment^2^, so the wastewater with lead contamination is required to treat below water quality standards before releasing to receive water for a safe environment.

Many conventional methods of coagulation–flocculation, chemical precipitation, oxidation–reduction, and ion exchange are used for eliminating heavy metals in wastewater; however, they have the disadvantages of incomplete heavy metal removals, high operating costs, and creating toxic sludges^3^. As a result, the alternative option for heavy metal removals which is an efficient method, easy operation, and suitable operating cost is an adsorption method. Moreover, this method also offers many available adsorbent choices depending upon the contaminated target ions, budget, and water quality requirement. This study will focus on waste peels because of their benefits as low-cost adsorbents used for improving water quality along with reducing waste peel volumes in case of waste management and disposal. The elimination of heavy metals in wastewater from various waste peels such as potato, papaya, cucumber, banana, and orange in 2013–2022 is demonstrated in Table 1. Among those adsorbents, orange peels are a good choice because it has good chemical properties of cellulose, pectic, hemicellulose, and lignin. In addition, the main functional groups of the hydroxyl group (–OH), a carboxyl group (–COOH), alkane (–CH_2_), and alkene (–CH_3_) in orange peels can adsorb lead in wastewater^4^. Although orange peels have good chemical properties for lead adsorption, they also need to improve in case of high lead concentration for industrial applications.

**Table 1:** The elimination of heavy metals in wastewater from various waste peels.

Waste peels	Heavy metal ions	Removal (%)	References
Potato	Pb (II)	–	^16^
Papaya	Pb (II)	97.54%	^17^
Pomelo	Pb (II)	–	^18^
Watermelon	Fe (II)	–	^19^
	Pb (II)	–	^19^
Cucumber	Cu (II)	–	^20^
	Pb (II)	–	^20^
	Pb (II)	93.50%	^21^
Garlic	Pb (II)	–	^22^
	Cu (II)	–	^22^
	Ni (II)	–	^22^
Pomegranate	Cd (II)	50.50%	^23^
	Cu (II)	80.00%	^23^
	Zn (II)	32.50%	^23^
Banana	Pb (II)	–	^24^
	Pb (II)	88.94%	^25^
	Pb (II)	–	^26^
	Cu (II)	–	^27^
	Cu (II)	–	^26^
	Cu (II)	99.79%	^25^
	Ni (II)	–	^24^
Orange	Cu (II)	80.00%	^28^
	Pb (II)	90.00%	^28^
	Cu (II)	–	^29^
	Cd (II)	–	^30^
	Pb (II)	–	^30^
	Hg (II)	–	^30^
	Cu (II)	86.27%	^31^
	Pb (II)	98.85%	^31^

Various modification methods of acid or base treatment, hydrothermal process, and modification with metal oxides to increase material adsorption capacity in many previous studies are reported in Table 2. Moreover, several types of metal oxides such as zinc oxide (ZnO), iron (II) oxide (Fe_3_O_4_), titanium dioxide (TiO_2_), magnesium oxide (MgO), iron (III) oxide-hydroxide, and geothite (FeO(OH)) are also used for improving the material ability for heavy metal removals in many studies^5–15^. Since iron (III) oxide-hydroxide offers high adsorbent efficiency for heavy metal adsorptions in many previous articles^5,6,9–12^, this study will improve the orange peel efficiency by modifying it with iron (III) oxide-hydroxide for lead adsorption. Therefore, this study attempts to synthesize orange peel powder materials with or without modification by iron (III) oxide-hydroxide, to compare their lead removal efficiencies through batch experiments, and verify whether adding metal oxide helped to improve material efficiency for lead adsorption.

**Table 2:** Several modification methods for improving the adsorbent capacity of various waste peels to eliminate heavy metals in wastewater.

Modifications	Waste peels	Heavy metal ions	Removal (%)	References
Activated carbon	Orange	Pb (II)	–	^32^
Biochar	Orange	Pb (II)	–	^33^
	Pomelo	Pb (II)	–	^34^
	Pomelo	Cu (II)	–	^34^
	Banana	Pb (II)	–	^35^
Acetic acid	Orange	Pb(II)	–	^36^
H_2_SO_4_	Dragon fruit	Pb(II)	92.92%	^37^
	Dragon fruit	Cd (II)	96.77%	^37^
	Rambutan	Pb(II)	97.87%	^37^
	Rambutan	Cd (II)	97.10%	^37^
	Passion fruit	Pb(II)	94.54%	^37^
	Passion fruit	Cd (II)	93.43%	^37^
H_2_SO_4_ and KMnO_4_	Pineapple	Cd (II)	–	^38^
	Pineapple	Pb(II)	–	^38^
HCl	Mexerica mandarin	Cu (II)	–	^39^
	Mexerica mandarin	Cd (II)	–	^39^
	Mexerica mandarin	Pb(II)	–	^39^
NaOH	Orange	Cu (II)	–	^40^
	Orange	Pb(II)	–	^40^
	Orange	Zn (II)	–	^40^
	Banana	Pb (II)	–	^27^
	Banana	Pb (II)	–	^41^
	Banana	Cu (II)	–	^27^
MgO	Watermelon	Pb (II)	–	^15^
TiO_2_	Pomegranate	As(III)	–	^7^
	Lemon	Ni (II)	78.00%	^42^
Al_2_O_3_	Orange	Ni (II)	–	^43^
	Orange	Cd (II)	–	^43^
	Banana	Cd (II)	99.80%	^44^
	Banana	Pb (II)	97.40%	^44^
	Lemon	Cd (II)	93.50%	^45^
CoFe_2_O_4_	Dragon fruit	Ni (II)	88.00%	^46^
Fe^2^^+^and Fe^3^^+^	Pomegranate	Pb (II)	–	^47^
FeCl_3_·6H_2_O	Pomelo	Pb (II)	–	^34^
	Pomelo	Cu (II)	–	^34^
Fe_3_O_4_	litchi	Pb (II)	–	^14^
	Yam	Hg (II)	–	^48^

This study aimed to synthesize orange peel powder (OP) and orange peel powder doped iron (III) oxide-hydroxide (OPF), to characterize their sizes of surface area, pore volumes, pore sizes, crystalline structures, surface morphologies, chemical elements, and chemical functional groups by Brunauer–Emmett–Teller (BET), X-Ray Diffractometer (XRD), Field Emission Scanning Electron Microscopy and Focus Ion Beam (FESEM-FIB) with Energy Dispersive X-Ray Spectrometer (EDX), and Fourier Transform Infrared Spectroscopy (FT-IR), and to examine their lead removal efficiencies by batch experiments. Their lead adsorption patterns and mechanisms were also studied by linear and nonlinear adsorption isotherms of Langmuir, Freundlich, Temkin, and Dubinin–Radushkevich models and kinetics of pseudo-first-kinetic, pseudo-second-kinetic, elovich, and intraparticle diffusion models. Finally, desorption experiments were investigated for the possibility of their reusable capacities.

## Materials and methods

### Raw material

Orange (*Citrus reticulata*) peels used in this study are wastes from an orange juice shop at the local market in Khon Kaen province, Thailand.

### Chemicals

All chemicals were analytical grades (AR) without purification before use. 95% Ethanol (C_2_H_5_OH), ferric chloride hexahydrate (FeCl_3_·6H_2_O) (LOBA, India), and sodium hydroxide (NaOH) (RCI Labscan, Thailand) were used for material synthesis, and lead nitrate (Pb(NO_3_)_2_) (QRëC, New Zealand) was used for preparing the synthetic wastewater. For the point of zero charge, 0.1 M hydrochloric acid (HCI) (RCI Labscan, Thailand), 0.1 M sodium chloride (NaCl) (RCI Labscan, Thailand), and 0.1 M NaOH were used. Finally, 1% HNO_3_ and 1% NaOH were used for pH adjustments.

### The material synthesis of OP and OPF

**Figure 1:**
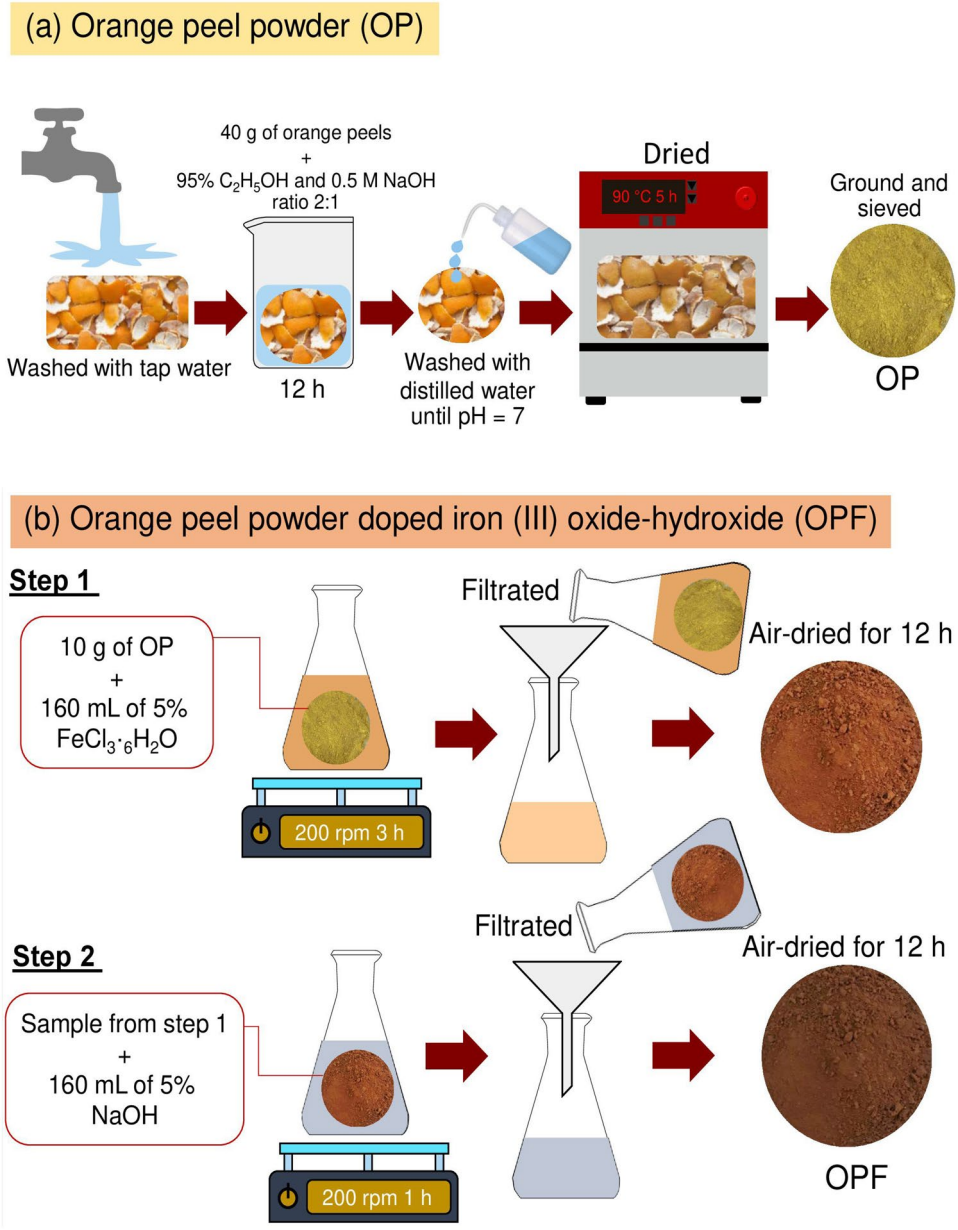
Material synthesis methods of (**a**) OP and (**b**) OPF.

Figure 1a,b is demonstrated the material synthesis methods of OP and OPF which are based on Threepanich and Praipipat^5^. The details were clearly explained below.

#### The material synthesis of OP

Firstly, orange peels were washed with tap water to eliminate contaminations. Next, 40 g of orange peels were soaked in the solution of 95% C_2_H_5_OH and 0.5 M NaOH in a ratio of 2:1 for 12 h. Then, they were washed with distilled water until the solution turned to a pH of 7. After that, they were dried in a hot air oven (Binder, FED 53, Germany) at 90 °C for 5 h. Then, they were ground and sieved in size of 125 µm. Finally, they were kept in a desiccator before use called orange peel powder (OP).

#### The material synthesis of OPF

Firstly, 10 g of OP were added to 500 mL of Erlenmeyer flask containing 160 mL of 5% FeCl_3_·6H_2_O. Then, they were mixed by an orbital shaker (GFL, 3020, Germany) of 200 rpm for 3 h. Next, they were filtrated and air-dried at room temperature for 12 h. After that, they were added to 500 mL of Erlenmeyer flask containing 160 mL of 5% NaOH, and they were mixed by an orbital shaker of 200 rpm for 1 h. Finally, they were filtered, air-dried at room temperature for 12 h, and kept in a desiccator before use called orange peel powder doped iron (III) oxide-hydroxide (OPF).

### Material characterizations of OP and OPF

The specific surface area, pore volumes, pore sizes, crystalline structures, surface morphologies, chemical compositions, and chemical functional groups of OP and OPF which were characterized by Brunauer–Emmett–Teller (BET) (Bel, Bel Sorp mini X, Japan) by isothermal nitrogen gas (N_2_) adsorption–desorption at 77.3 K and degas temperature of 80 ℃ for 6 h, X-Ray Diffractometer (XRD) (PANalytical, EMPYREAN, United Kingdom) in a range of 2θ = 5°–80°, Field Emission Scanning Electron Microscopy and Focus Ion Beam (FESEM-FIB) with Energy Dispersive X-Ray Spectrometer (EDX) (FEI, Helios NanoLab G3 CX, USA which the samples placed on aluminum stubs with gold-coating for 4 min using a 108 auto Sputter Coater with thickness controller MTM-20 model (Cressington, Ted Pella Inc, USA) by analyzing at 10 kV accelerating voltage, and Fourier Transform Infrared Spectroscopy (FT-IR) (Bruker, TENSOR27, Hong Kong) in a range of 600–4000 cm^−1^ with a resolution of 4 cm^−1^ and 16 scans over the entire covered range.

### The point of zero charges of OP and OPF

The points of zero charges of OP and OPF for lead adsorptions were studied referred from the study of Praipipat et al.^10^ by using 0.1 M NaCl solutions with pH values from 2 to 12 which 0.1 M HCl and 0.1 M NaOH were used for pH adjustments. Firstly, 0.1 g of each adsorbent material (OP or OPF) was added to 250 mL Erlenmeyer flasks containing 50 mL of each 0.1 M NaCl solution. Then, it was shaken by an orbital shaker (GFL, 3020, Germany) at room temperature at 150 rpm for 24 h. After that, the final pH value of the sample solution was measured by a pH meter (Mettler Toledo, SevenGo with InLab 413/IP67, Switzerland) and ∆pH (pH_final_ − pH_initial_) was calculated. A point that is the crosses line of ∆pH versus pH_initial_ equal to zero is the value of the point of zero charges (pH_pzc_)^49^.

### Batch experiments

A series of batch experiments were designed to investigate lead adsorption efficiencies of OP and OPF with different values of dose (1 to 6 g), contact time (1 to 6 h), pH (1, 3, 5, 7, 9, 11), and concentration (10 to 70 mg/L) with the control condition was the initial lead concentration of 50 mg/L, a sample volume of 100 mL, pH 5, a shaking speed of 200 rpm, and a temperature of 25 ℃. The optimum value was selected from the lowest value of each factor with the highest lead removal efficiency, and that value was applied to the next affecting factor study. Lead concentrations are measured by the Atomic Adsorption Spectrophotometer (AAS) (PerkinElmer, PinAAcle 900 F, USA), and triplicate experiments were conducted to confirm the results. Lead removal efficiency in the percentage (%) is calculated by following Eq. (1)

1 $${\text{Lead removal efficiency}}\left( \% \right) = \, (C_{0} - C_{{\text{e}}} )/C_{0} \times { 1}00$$

where *C*_0_ is the initial lead concentration (mg/L), and *C*_e_ is the equilibrium of lead concentration in the solution (mg/L).

### Adsorption isotherms

Adsorption isotherms are designed to investigate the adsorption patterns of OP and OPF by using various adsorption models of linear and nonlinear Langmuir, Freundlich, Temkin, and Dubinin–Radushkevich. Graphs of linear Langmuir, Freundlich, Temkin, and Dubinin–Radushkevich isotherms were plotted by *C*_e_/*q*_e_ versus *C*_*e*_, log *q*_e_ versus log *C*_e_, *q*_e_ versus ln *C*_*e*_, and ln *q*_e_ versus *ε*^2^, respectively whereas graphs of their nonlinear were plotted by *q*_e_ versus *C*_e_. Their adsorption models are calculated by Eqs. (2)–(9)^50–53^:

Langmuir isotherm:

2 $${\text{Linear:}}\,\, C_{{\text{e}}} /q_{{\text{e}}} = 1/q_{{\text{m}}} K_{{\text{L}}} + C_{{\text{e}}} /q_{{\text{m}}}$$

3 $${\text{Nonlinear:}}\,\, q_{e} = q_{{\text{m}}} K_{{\text{L}}} C_{{\text{e}}} /{1} + K_{{\text{L}}} C_{{\text{e}}}$$

Freundlich isotherm:

4 $${\text{Linear:}}\,\, {\text{log}}q_{{\text{e}}} = {\text{log}}K_{{\text{F}}} + {1}/n{\text{log}}C_{{\text{e}}}$$

5 $${\text{Nonlinear:}}\,\,q_{{\text{e}}} = K_{{\text{F}}} C_{{\text{e}}}^{{{1}/n}}$$

Temkin isotherm:

6 $${\text{Linear:}}\,\, q_{e} = RT/b_{{\text{T}}} {\text{ln}}A_{{\text{T}}} + RT/b_{{\text{T}}} {\text{ln}}C_{{\text{e}}}$$

7 $${\text{Nonlinear:}}\,\, q_{{\text{e}}} = RT/b_{{\text{T}}} {\text{ln}}A_{{\text{T}}} C_{{\text{e}}}$$

Dubinin–Radushkevich isotherm:

8 $${\text{Linear:}}\,\, {\text{ln}}q_{{\text{e}}} = {\text{ ln}}q_{{\text{m}}} {-}K_{{{\text{DR}}}} \varepsilon^{{2}}$$

9 $${\text{Nonlinear:}}\,\, q_{e} = q_{{\text{m}}} {\text{exp}}( - K_{{{\text{DR}}}} \varepsilon^{{2}} )$$

where *C*_e_ is the equilibrium of lead concentration (mg/L), *q*_e_ is the amount of adsorbed lead on OP or OPF (mg/g), *q*_m_ is indicated the maximum amount of lead adsorption on OP or OPF (mg/g), *K*_L_ is the adsorption constant (L/mg). *K*_F_ is the constant of adsorption capacity (mg/g) (L/mg)^1/n^, and 1/*n* is the constant depicting the adsorption intensity. *R* is the universal gas constant (8.314 J/mol K), *T* is the absolute temperature (K), *b*_T_ is the constant related to the heat of adsorption (J/mol), and *A*_T_ is the equilibrium binding constant corresponding to the maximum binding energy (L/g). *q*_m_ is the theoretical saturation adsorption capacity (mg/g), *K*_DR_ is the activity coefficient related to mean adsorption energy (mol^2^/J^2^), and *ε* is the Polanyi potential (J/mol).

For adsorption isotherm experiments, 4 g of OP or 3 g of OPF were added to 250 mL Erlenmeyer flasks with variable lead concentrations from 10 to 70 mg/L. The control condition of OP or OPF was a sample volume of 100 mL, a shaking speed of 200 rpm, pH 5, a temperature of 25 °C, and a contact time of 6 h.

### Adsorption kinetics

Adsorption kinetics are used for studying adsorption rates and mechanisms of OP and OPF which various kinetic models of linear and nonlinear pseudo-first-order kinetic, pseudo-second-order kinetic, elovich, and intraparticle diffusion were applied. Graphs of linear pseudo-first-order, pseudo-second-order, elovich, and intraparticle diffusion models were plotted by ln (*q*_e_ − *q*_t_) versus time (*t*), *t*/*q*_t_ versus time (*t*), *q*_t_ versus ln *t*, and *q*_t_ versus time (*t*^0.5^), respectively whereas their nonlinear graphs were plotted by the capacity of lead adsorbed by adsorbent materials at the time (*q*_t_) versus time (*t*). Their adsorption kinetic equations are calculated by Eqs. (10)–(16)^54–57^:

Pseudo-first-order kinetic model:

10 $${\text{Linear:}}\,\,{\text{ln }}\left( {q_{{\text{e}}} - q_{{\text{t}}} } \right) = {\text{ln}}q_{{\text{e}}} {-} \, k_{{1}} t$$

11 $${\text{Nonlinear:}}\,\, q\mathrm{t} = q\mathrm{e}\left(1-{e}^{{-k}_{1}t}\right)$$

Pseudo-second-order kinetic model:

12 $${\text{Linear:}}\,\,t/q_{{\text{t}}} = {1}/k_{{2}} q_{{\text{e}}}^{{2}} + \, \left( {t/q_{{\text{e}}} } \right)$$

13 $${\text{Nonlinear:}}\,\, q_{{\text{t}}} = k_{{2}} q_{{\text{e}}}^{{2}} t/\left( {{1} + \, q_{{\text{e}}} k_{{2}} t} \right)$$

Elovich model:

14 $${\text{Linear:}}\,\, q_{t} = { 1}/\beta {\text{ln}}\alpha \beta + { 1}/\beta {\text{ln}}t$$

15 $${\text{Nonlinear:}}\,\,q_{t} = \beta {\text{ln}}t + \beta {\text{ln}}\alpha$$

Intraparticle diffusion model:

16 $${\text{Linear and nonlinear:}}\,\, q_{{\text{t}}} = k_{{\text{i}}} t^{{0.{5}}} + C_{{\text{i}}}$$

where *q*_e_ is the amount of adsorbed lead on adsorbent materials (mg/g), *q*_t_ is the amount of adsorbed lead at the time (*t*) (mg/g), *k*_1_ is a pseudo-first-order rate constant (min^−1^), and *k*_2_ is a pseudo-second-order rate constant (g/mg∙min). *α* is the initial adsorption rate (mg/g/min) and *β* is the extent of surface coverage (g/mg). *k*_i_ is the intraparticle diffusion rate constant (mg/g∙min^0.5^) and *C*_i_ is the constant that gives an idea about the thickness of the boundary layer (mg/g).

For adsorption kinetic experiments, 40 g of OP or 30 g of OPF were added to 1000 mL of breaker with the lead concentration of 50 mg/L. The control condition of OP and OPF was a sample volume of 1000 mL, a shaking speed of 200 rpm, pH 5, a temperature of 25 °C, and a contact time of 8 h.

### Desorption experiments

The five adsorption–desorption cycles are designed for desorption experiments of OP and OPF for lead adsorption to investigate the possible material reusability. The saturated OP or OPF from the adsorption process was added to 500 mL of Erlenmeyer flask containing 200 mL of 0.5 M HNO_3_ solution, and it was shaken by an incubator shaker (New Brunswick, Innova 42, USA) at 200 rpm for 6 h. After that, it was washed with deionization water and dried at room temperature. Then, OP or OPF is ready for the next adsorption cycle. The desorption efficiency in percentage is calculated by following Eq. (17):

17 $${\text{Desorption }}\left( \% \right) \, = \, \left( {q_{{\text{d}}} /q_{a} } \right) \, \times { 1}00$$

where *q*_*d*_ is the amount of lead desorbed (mg/mL) and *q*_a_ is the amount of lead adsorbed (mg/mL).

## Result and discussion

### The physical characteristics of OP and OPF

**Figure 2:**
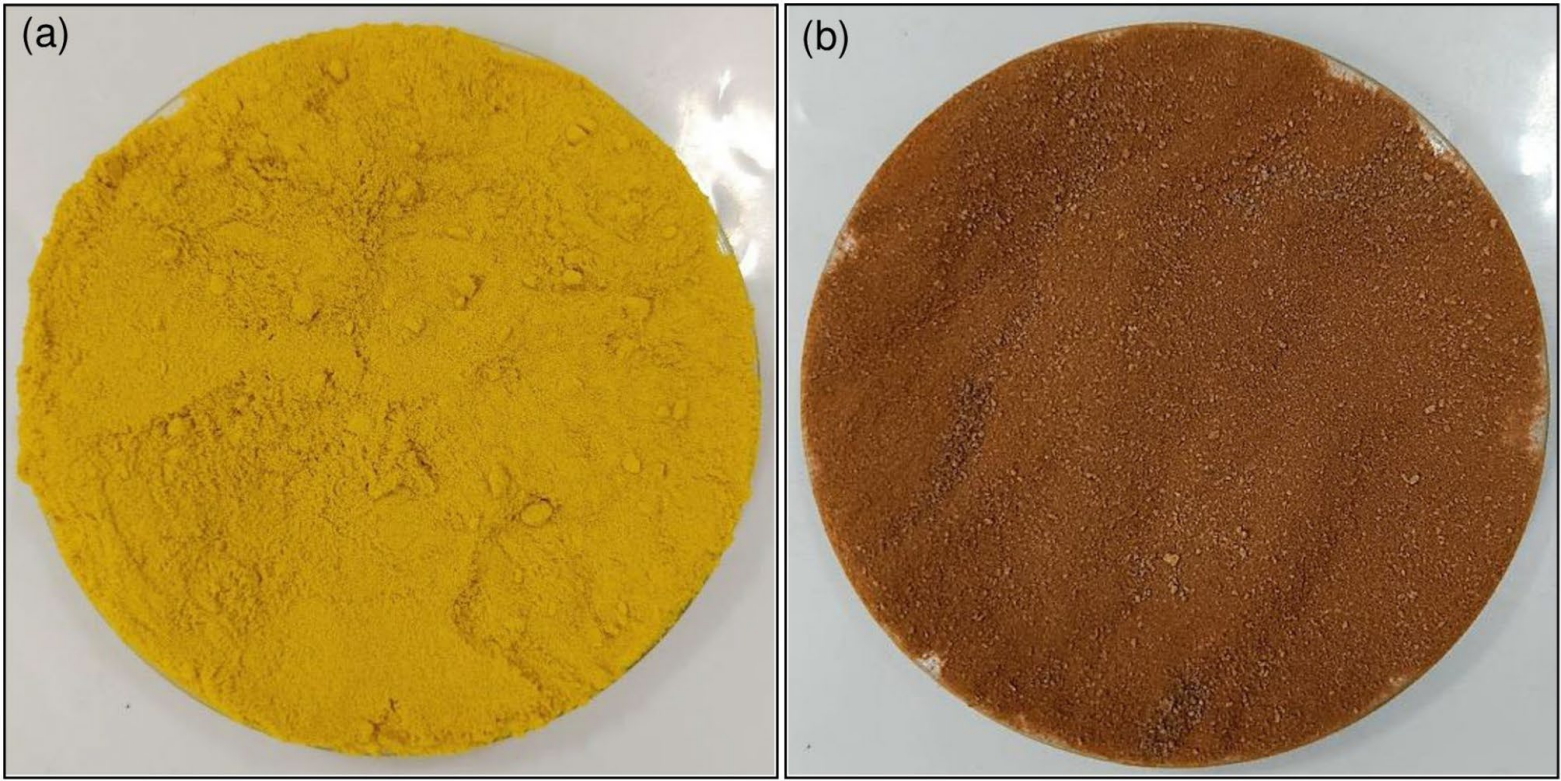
The physical characteristics of (**a**) OP and (**b**) OPF.

The physical characteristics of OP and OPF are demonstrated in Fig. 2a,b. OP was a yellow color powder shown in Fig. 2a while OPF was an iron-rust color powder corresponding to a color of iron (III) oxide-hydroxide color shown in Fig. 2b.

### Material characterizations of OP and OPF

#### BET

Brunauer–Emmett–Teller (BET) technique with N_2_ adsorption–desorption isotherm at 77.3 K and degas temperature of 80 °C for 6 h was used for determining the specific surface area, pore volume, and pore diameter size of OP and OPF. The results of the surface area and pore volume are reported by Brunauer–Emmett–Teller (BET) method, whereas the pore size is reported by Barrett-Joyner-Halenda (BJH) method shown in Table 3. For OP, its specific surface, pore volume, and pore size were 0.431 m^2^/g, 0.099 cm^3^/g, and 4.462 nm, respectively. The specific surface, pore volume, and pore size of OPF were 0.896 m^2^/g, 0.206 cm^3^/g, and 2.575 nm, respectively. As a result, OPF had a higher specific surface area and pore volume than OP, whereas OPF had a smaller pore size than OP. Thus, the addition of iron (III) oxide-hydroxide into OPF affected to increase its specific surface area and pore volume and decrease its pore size similar reported by previous studies^6,10–12^. Since the pore sizes of OP and OPF were in a range of 2–50 nm, they were classified to be as mesoporous material following the classification by the International Union of Pure and Applied Chemistry (IUPAC)^58^.

**Table 3:** The specific surface area, pore volume, and pore size of OP and OPF.

Materials	Surface area* (m^2^/g)	Pore volume** (cm^3^/g)	Pore size*** (nm)
OP	0.431	0.099	4.462
OPF	0.896	0.206	2.575

*BET specific surface area.

**Total pore volume.

***Pore diameter size.

#### XRD

**Figure 3:**
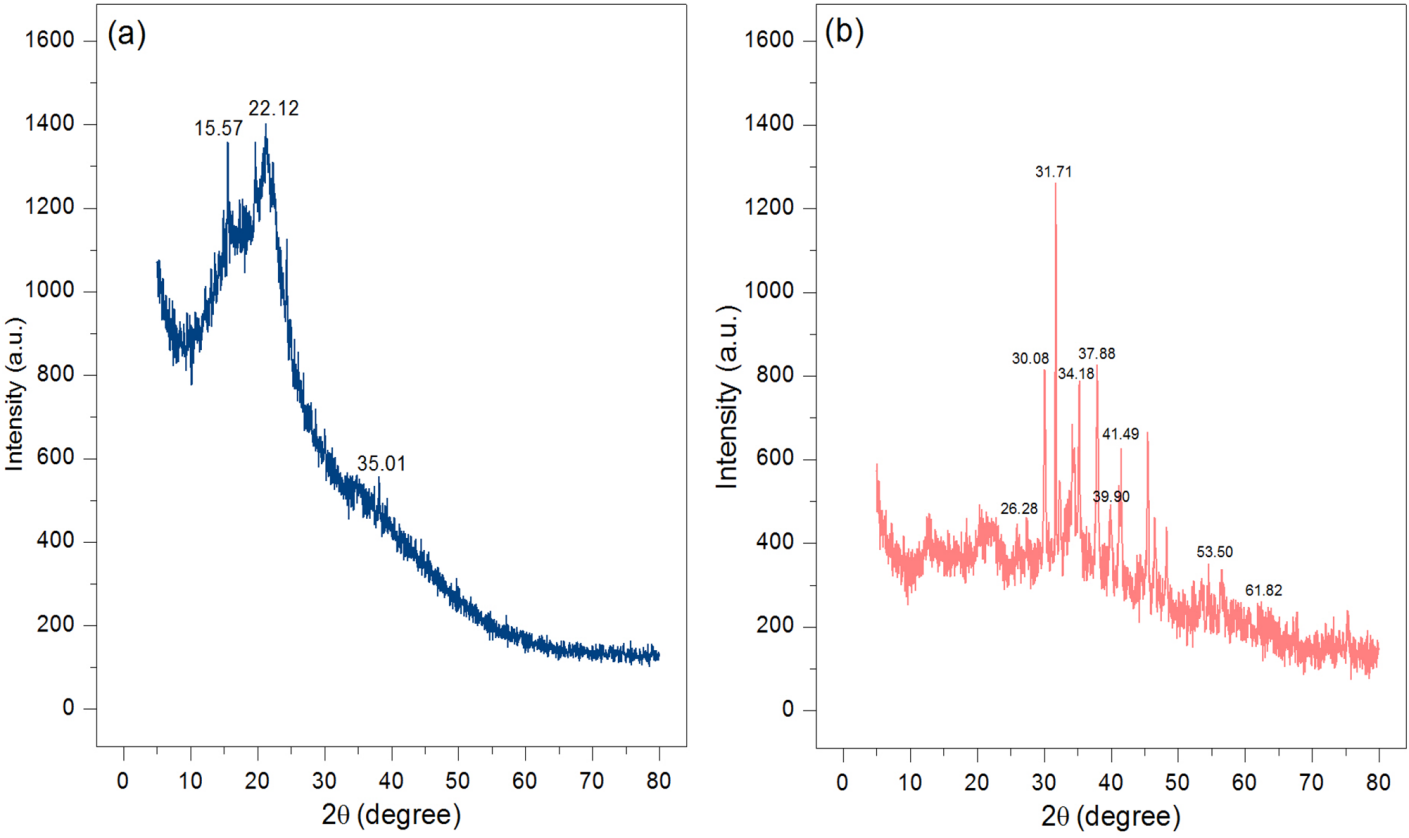
The crystalline structures of (**a**) OP and (**b**) OPF.

X-Ray Diffractometer (XRD) was used for characterizing the crystalline structures of OP and OPF, and their results are shown in Fig. 3a,b. OP and OPF were semi-crystalline structures that presented the specific cellulose peaks at 2θ values of approximately 15.57°, 22.71°, and 35.01°^59^. In addition, the specific iron (III) oxide-hydroxide peaks at 2θ values of approximately 26.28°, 30.08°, 31.71°, 34.18°, 37.88°, 39.90°, 41.49°, 53.50°, and 61.82° following JCPDS No. 29-0713^60^ were found in OPF, so it could confirm the addition of iron (III) oxide-hydroxide into OPF.

#### FESEM-FIB

**Figure 4:**
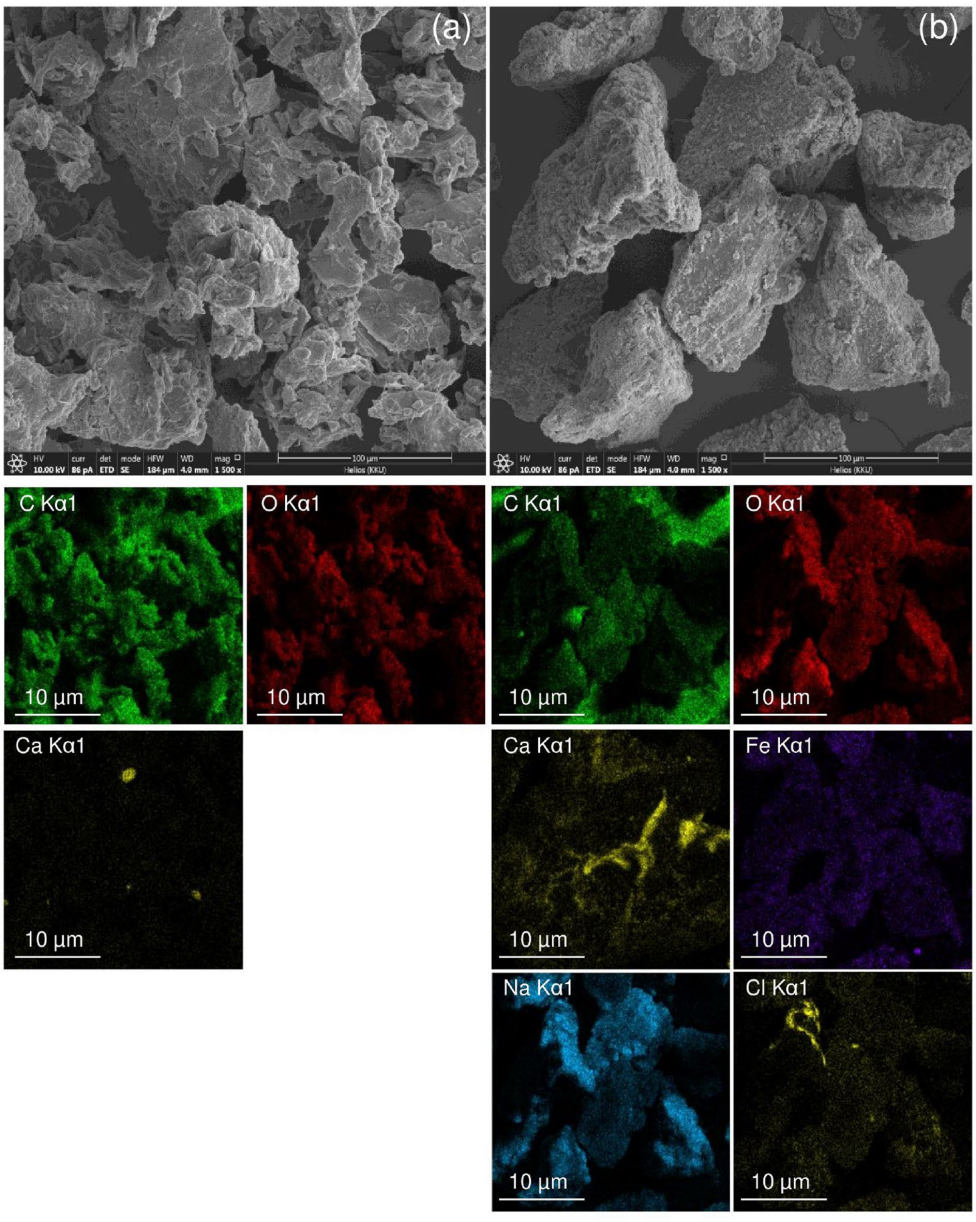
The surface morphologies and dispersions of chemical elements by elemental mapping of (**a**) OP and (**b**) OPF.

Field Emission Scanning Electron Microscopy and Focus Ion Beam (FESEM-FIB) at × 1500 magnification with 100 µm were used for investigating the surface morphologies of OP and OPF. Their results and the dispersions of chemical elements by elemental mapping are illustrated in Fig. 4a,b. For OP, it was irregular and porous surfaces. OPF had the same structure and morphology surface as the OP, but its size was larger than the OP at the same magnification.

#### EDX

Energy Dispersive X-Ray Spectrometer (EDX) was used for determining the chemical compositions of OP and OPF, and their results are demonstrated in Table 4. Carbon (C), oxygen (O), and calcium (Ca) were the main chemical elements in both materials similar found in other studies^31,40^, whereas iron (Fe), sodium (Na), and chloride (Cl) were only found in OPF. Fe, Na, and Cl might be from using chemicals of ferric chloride hexahydrate (FeCl_3_·6H_2_O) and sodium hydroxide (NaOH) for synthesizing OPF.

**Table 4:** The chemical compositions of OP and OPF.

Materials	Chemical compositions (%wt)					
	C	O	Ca	Fe	Na	Cl
OP	51.4	47.8	0.8	–	–	–
OPF	42.2	42.1	0.4	14.8	0.4	0.1

#### FT-IR

**Figure 5:**
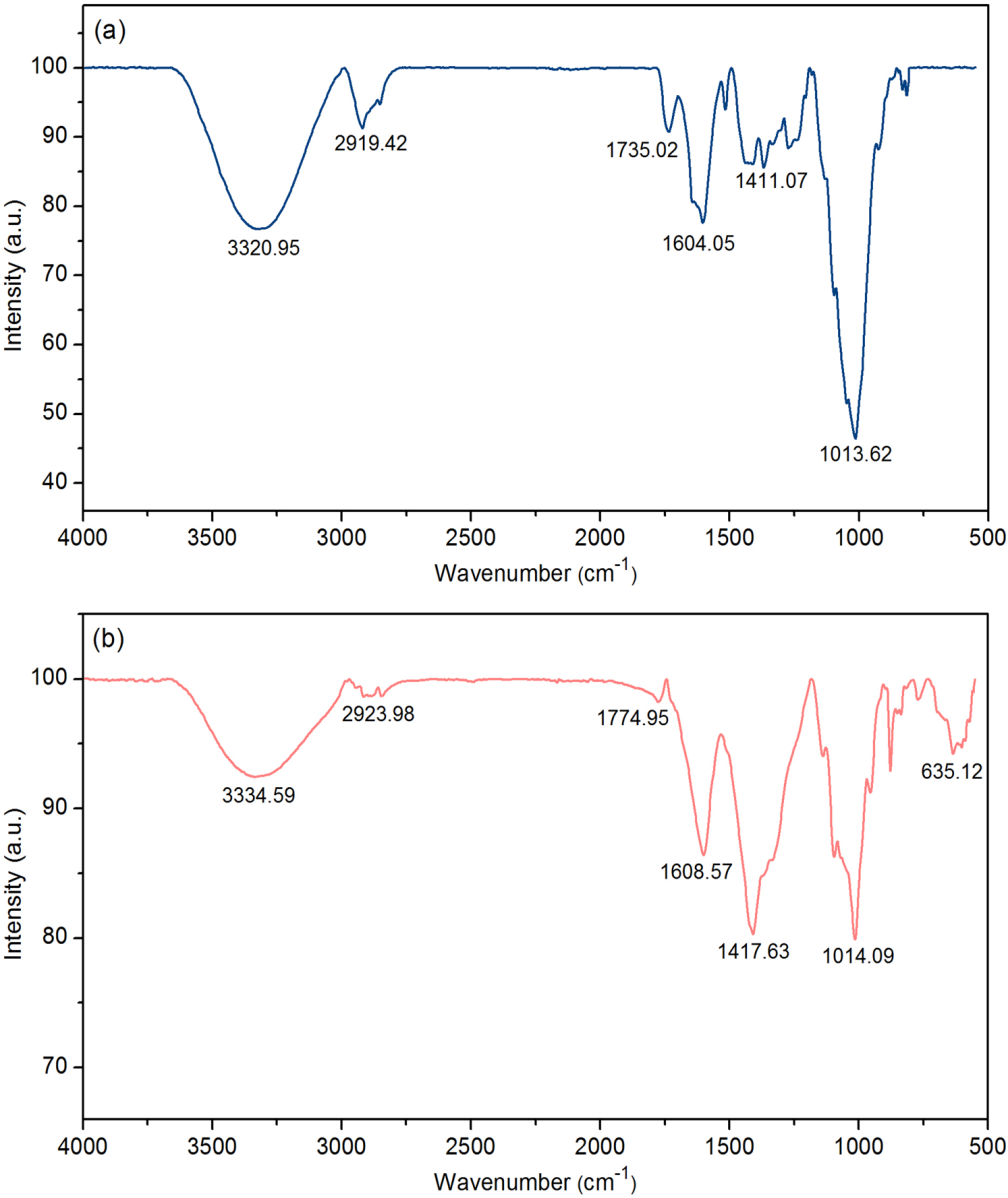
The chemical functional groups of (**a**) OP and (**b**) OPF.

Fourier Transform Infrared Spectroscopy (FT-IR) was used for identifying the chemical functional groups of OP and OPF, and their FT-IR spectra are demonstrated in Fig. 5a,b. O–H, C–H, C=C, C–O, C=O, and –COOH were the main functional groups of both materials whereas Fe–O was only found in OPF which might be from the addition of iron (III) oxide-hydroxide^61^. For O–H, it represented the stretching of the alcohol, phenol, and hydroxyl group in lignin or hemicellulose, and C–H was the stretching of the aliphatic nature of orange peels^62^. C=C demonstrated the double bond in aromatic rings, and C–O demonstrated the aliphatic chains of –CH_2_ and –CH_3_ in the basic structure of lignin or cellulose^63^. C=O and –COOH presented the carboxyl groups^59^. For OP, it found the stretching of O–H at 3320.95 cm^−1^, C–H at 2919.42 cm^−1^, stretching of C=C at 1604.05 cm^−1^, stretching of C–O at 1013.62 cm^−1^, stretching of C=O at 1735.02 cm^−1^, and stretching of –COOH at 1411.07 cm^−1^ shown in Fig. 5a. For OPF, it found the stretching of O–H at 3334.59 cm^−1^, C–H at 2923.98 cm^−1^, stretching of C=C at 1608.57 cm^−1^, stretching of C–O at 1014.09 cm^−1^, stretching of C=O at 1774.95 cm^−1^, stretching of –COOH at 1417.63 cm^−1^, and stretching of Fe–O at 635.12 cm^−1^ shown in Fig. 5b. As a result, the addition of iron (III) oxide-hydroxide affected to the higher stretching of all main functional groups of OPF more than OP which they might result to increase lead adsorption of OPF more than OP.

#### The point of zero charges (pH_pzc_) of OP and OPF

**Figure 6:**
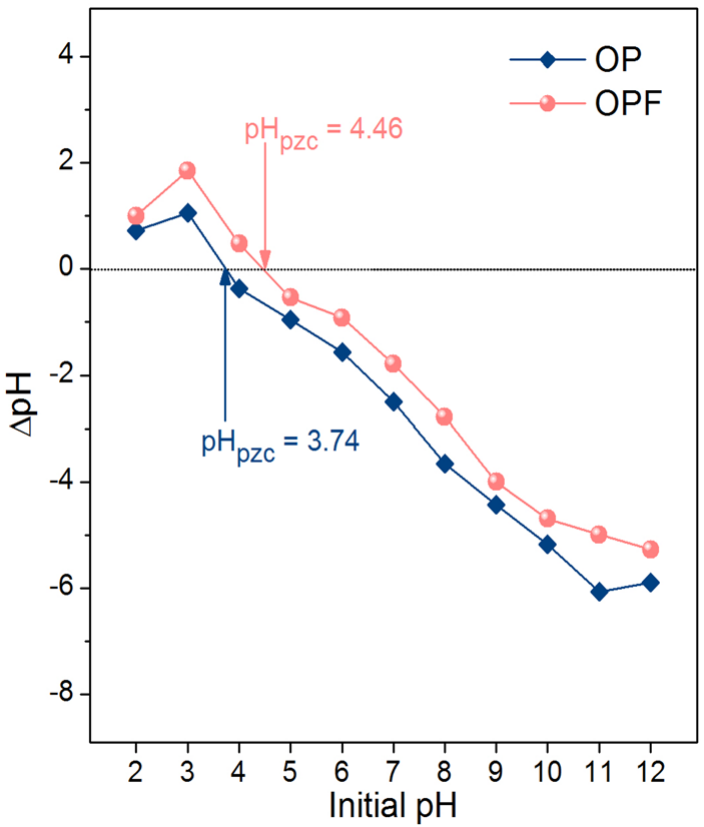
The point of zero charges of OP and OPF.

The point of zero charge (pH_pzc_) refers to a pH value at the net charge equal to zero of the adsorbent which uses for determining which pH value is good for lead adsorption by material. In Fig. 6, the pH_pzc_ of OP and OPF were 3.74 and 4.46, so the addition of iron (III) oxide-hydroxide affected the increase of pH_pzc_ similar reported by previous studies^9–11^. The pH of the solution and the pH_pzc_ are normally used for considering lead adsorption by material which the high lead adsorption should be found at the pH of solution higher than pH_pzc_ (pH_solution_ > pH_pzc_) because of occurring negatively charged of material. In addition, many previous studies reported high lead adsorption at a pH solution higher than pH 4^5,6,8,64^.

### Batch experiments

#### The effect of dose

**Figure 7:**
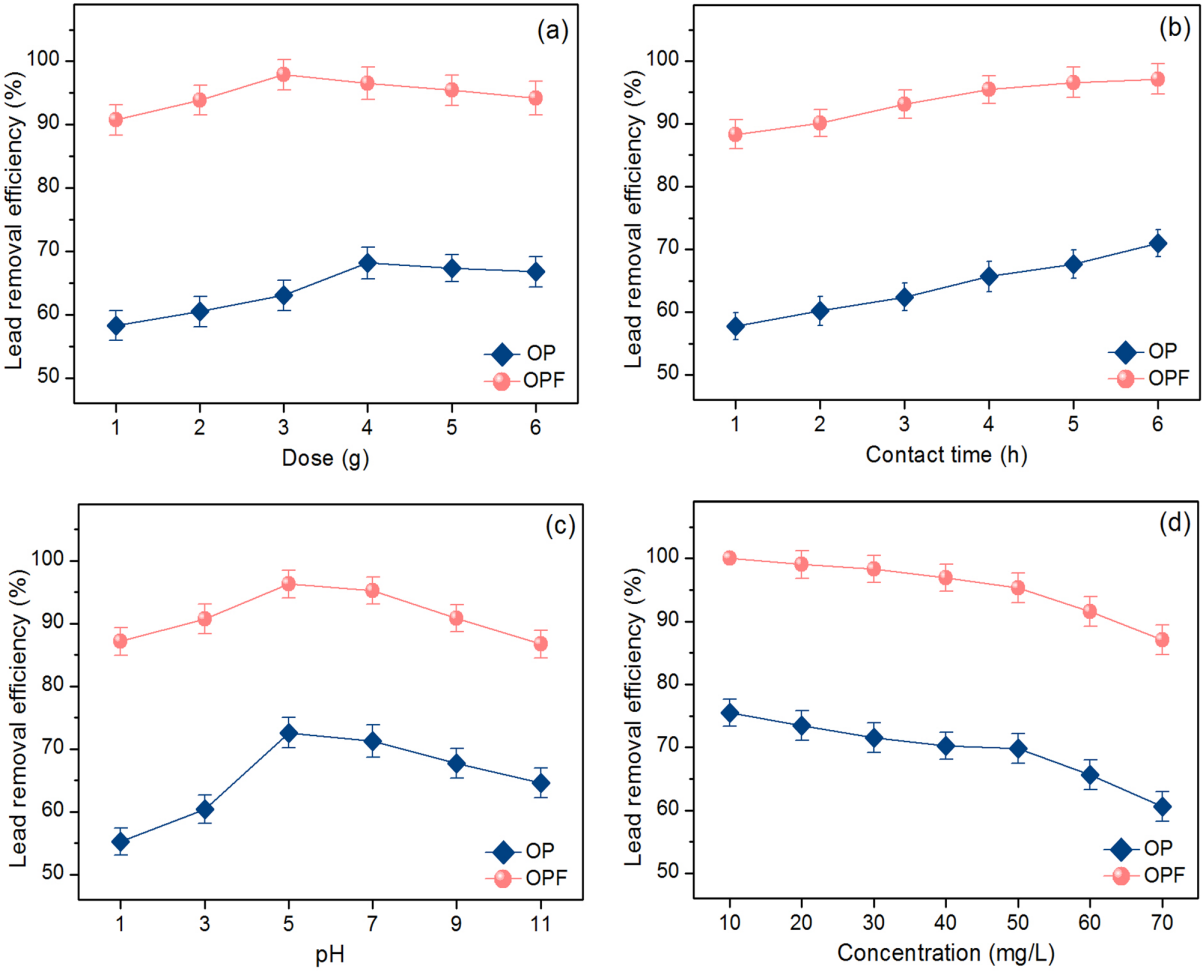
Batch experiments on the effects of (**a**) dose, (**b**) contact time, (**c**) pH, and (**d**) concentration of OP and OPF.

The effect of dose was investigated lead removal efficiencies by varying six different dosages from 1 to 6 g of OP and OPF with the control condition of the lead concentration of 50 mg/L, a sample volume of 100 mL, a contact time of 5 h, pH 5, a temperature of 25 ℃, and a shaking speed of 200 rpm. The results are demonstrated in Fig. 7a. Lead removal efficiencies of both materials were increased with the increase in dosage which might be the increase of the active sites of materials^8^. Their highest lead removal efficiencies were 68.16% at 4 g for OP and 97.86% at 3 g for OPF. Therefore, they were optimum doses of OP and OPF that were used for studying the contact time effect.

#### The effect of contact time

The effect of contact time was investigated lead removal efficiencies by varying six different contact times from 1 to 6 h of OP and OPF with the control condition of the lead concentration of 50 mg/L, a sample volume of 100 mL, pH 5, a temperature of 25 ℃, a shaking speed of 200 rpm, and the optimum dose. The results are demonstrated in Fig. 7b. Lead removal efficiencies of both materials were increased with the increase of contact time similar to the dose effect, and the saturated lead adsorption on the material is generally found at the highest lead removal efficiency^9^. Their highest lead removal efficiencies of both materials were 6 h at 70.95% and 97.13% for OP and OPF. Therefore, they were the optimum contact time of OP and OPF which were used for studying the pH effect.

#### The effect of pH

The effect of pH was investigated lead removal efficiencies by varying six pH values of 1, 3, 5, 7, 9, and 11 as respective pH conditions of acid, neutral, and base of OP and OPF with the control condition of the lead concentration of 50 mg/L, a sample volume of 100 mL, a temperature of 25 ℃, a shaking speed of 200 rpm, and the optimum dose and contact time. The results are demonstrated in Fig. 7c. Lead removal efficiencies of both materials were increased with the increase of pH values from 1 to 5, then they were decreased. Their highest lead removal efficiencies were found at pH 5 with lead removal at 72.60% and 96.27% for OP and OPF. This result corresponded to other previous studies that reported the highest lead removal efficiency at pH > 4 relating to the results of pH_pzc_ of OP and OPF^5,6,8,64^. Therefore, pH 5 was the optimum pH of OP and OPF which were used for studying the concentration effect.

#### The effect of concentration

The effect of concentration was investigated lead removal efficiencies by varying seven different concentrations from 10 to 70 mg/L of OP and OPF with the control condition of a sample volume of 100 mL, a temperature of 25 ℃, a shaking speed of 200 rpm, and the optimum dose, contact time, and pH. The results are demonstrated in Fig. 7d. Lead removal efficiencies of both materials were decreased with the increasing of concentrations which might be from the active sites of them did not enough to caught up with lead ions similarly reported by other studies^5,8,64^. For the lead concentration of 50 mg/L, lead removal efficiencies of OP and OPF were 69.78% and 95.29%, and OPF demonstrated a higher lead removal efficiency than OP.

In conclusion, 4 g, 6 h, pH 5, 50 mg/L and 3 g, 6 h, pH 5, 50 mg/L were the optimum conditions in dose, contact time, pH, and concentration of OP and OPF. As a result, adding iron (III) oxide-hydroxide helped to improve material efficiency for lead adsorption similar reported by previous studies^5,6^, and OPF was recommended to be applied for lead removal in future industrial applications.

### Adsorption isotherms

**Figure 8:**
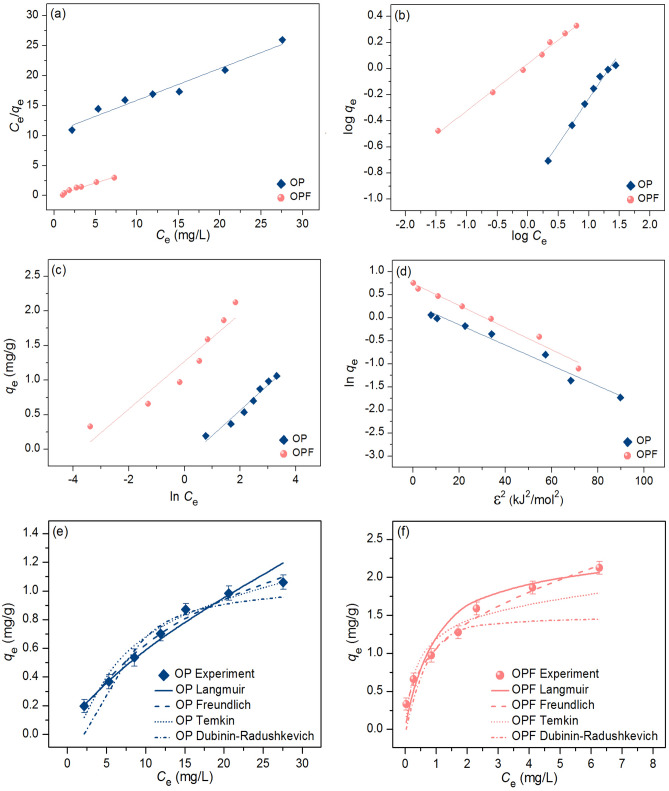
Graphs of (**a**) linear Langmuir, (**b**) linear Freundlich, (**c**) linear Temkin, (**d**) linear Dubinin–Radushkevich, and (**e, f**) nonlinear adsorption isotherms of OP and OPF for lead adsorptions.

The adsorption patterns of OP and OPF for lead adsorptions were investigated through linear and nonlinear models of Langmuir, Freundlich, Temkin, and Dubinin–Radushkevich models. For linear models, Langmuir, Freundlich, Temkin, and Dubinin–Radushkevich isotherms were plotted by *C*_e_/*q*_e_ versus *C*_e_, log *q*_e_ versus log *C*_e_, *q*_e_ versus ln *C*_e_, and ln *q*_e_ versus *ε*^2^, respectively. For nonlinear models, all isotherms were plotted by* C*_e_ versus *q*_e._ The plotting graphs are demonstrated in Fig. 8a–f, and the equilibrium isotherm parameters are illustrated in Table 5.

**Table 5:** The comparison of linear and nonlinear isotherm parameters for lead adsorptions on OP and OPF.

Regression method	Isotherm model	Parameter	OP	OPF
Linear	Langmuir	*q*_m_ (mg/g)	1.879	2.303
		*K*_L_ (L/mg)	0.050	1.179
		*R*^2^	0.964	0.969
	Freundlich	1*/n*	0.697	0.363
		*K*_F_ (mg/g) (L/mg)^1/n^	0.119	1.093
		*R*^2^	0.987	0.995
	Temkin	*b*_T_ (J/mol)	6808.072	7224.771
		*A*_T_ (L/g)	0.638	40.628
		*R*^2^	0.960	0.910
	Dubinin–Radushkevich	*q*_*m*_ (mg/g)	0.794	1.445
		*K*_DR_ (mol^2^/J^2^)	1.679	0.022
		*E* (kJ/mol)	0.546	4.757
		*R*^2^	0.941	0.781
Nonlinear	Langmuir	*q*_m_ (mg/g)	1.895	2.341
		*K*_L_ (L/mg)	0.054	1.185
		*R*^2^	0.968	0.971
		*R*^2^_adj_	0.962	0.965
		RMSE	0.050	0.208
	Freundlich	1*/n*	0.699	0.373
		*K*_F_ (mg/g) (L/mg)^1/n^	0.122	1.087
		*R*^2^	0.989	0.993
		*R*^2^_adj_	0.987	0.991
		RMSE	0.253	0.060
	Temkin	*b*_T_ (J/mol)	6814.794	7229.886
		*A*_T_ (L/g)	0.641	42.672
		*R*^2^	0.958	0.919
		*R*^2^_adj_	0.950	0.903
		RMSE	0.081	0.257
	Dubinin–Radushkevich	*q*_*m*_ (mg/g)	0.782	1.464
		*K*_DR_ (mol^2^/J^2^)	1.680	0.082
		*E* (kJ/mol)	0.574	4.685
		*R*^2^	0.946	0.786
		*R*^2^_adj_	0.935	0.743
		RMSE	0.117	0.415

For linear models, the Langmuir maximum adsorption capacities (*q*_m_) of OP and OPF were 1.879 and 2.303 mg/g, and Langmuir adsorption constants (*K*_L_) of OP and OPF were 0.050 and 1.179 L/mg. For Freundlich isotherm, the 1/*n* values of OP and OPF were 0.697 and 0.363. Freundlich adsorption constants (*K*_F_) of OP and OPF were 0.119 and 1.093 (mg/g) (L/mg)^1/n^. For Temkin isotherm, *b*_T_ values of OP and OPF were 6808.072 and 7224.771 (J/mol), and *A*_T_ values of OP and OPF were 0.638 and 40.628 L/g. For the Dubinin–Radushkevich model, the maximum adsorption capacities (*q*_m_) of OP and OPF were 0.794 and 1.445 mg/g, and the activity coefficient (*K*_DR_) values of OP and OPF were 1.679 and 0.022 mol^2^/J^2^, respectively. The adsorption energy (*E*) values of OP and OPF were 0.546 and 4.757 kJ/mol. *R*^2^ values of OP and OPF on Langmuir and Freundlich models were 0.964, 0.969 and 0.987, 0.995, respectively. In addition, *R*^2^ values of OP and OPF on Temkin and Dubinin–Radushkevich models were 0.960, 0.910 and 0.941, 0.781, respectively.

For nonlinear models, the Langmuir maximum adsorption capacities (*q*_m_) of OP and OPF were 1.895 and 2.341 mg/g, and Langmuir adsorption constants (*K*_L_) of OP and OPF were 0.054 and 1.185 L/mg. For Freundlich isotherm, the 1/*n* values of OP and OPF were 0.699 and 0.373. Freundlich adsorption constants (*K*_F_) of OP and OPF were 0.122 and 1.087 (mg/g) (L/mg)^1/n^. For Temkin isotherm, *b*_T_ values of OP and OPF were 6814.794 and 7229.886 (J/mol), and *A*_T_ values of OP and OPF were 0.641 and 42.672 L/g. For the Dubinin–Radushkevich model, the maximum adsorption capacities (*q*_m_) of OP and OPF were 0.782 and 1.464 mg/g, and the activity coefficient (*K*_DR_) values of OP and OPF were 1.680 and 0.082 mol^2^/J^2^, respectively. The adsorption energy (*E*) values of OP and OPF were 0.574 and 4.685 kJ/mol. *R*^2^ values of OP and OPF on Langmuir and Freundlich models were 0.968, 0.971 and 0.989, 0.993, respectively. In addition, *R*^2^ values of OP and OPF on Temkin and Dubinin–Radushkevich models were 0.958, 0.919 and 0.946, 0.786, respectively. Moreover, *R*^2^_adj_ of OP and OPF in nonlinear Langmuir and Freundlich models were 0.962, 0.965 and 0.987, 0.991, respectively. *R*^2^_adj_ of OP and OPF in nonlinear Temkin and Dubinin–Radushkevich models were 0.950, 0.903 and 0.935, 0.743, respectively.

For *R*^2^ value consideration, since *R*^2^ values of OP and OPF in both linear and nonlinear Freundlich models were higher than Langmuir, Temkin, and Dubinin–Radushkevich models, their adsorption patterns corresponded to Freundlich isotherm relating to physiochemical adsorption. Therefore, it recommends plotting isotherm graphs in both linear and nonlinear models to confirm the results and protect against data mistranslation^65–67^.

Moreover, the comparison of the maximum adsorption capacity (*q*_m_) value of waste peel adsorbents for lead adsorption is illustrated in Table 6. The orange peel powder doped iron (III) oxide-hydroxide (OPF) demonstrated a higher *q*_m_ value than the pomelo, banana, and lemon peels^5,18,68^ whereas the orange peel powder (OP) was a lower *q*_m_ value than all studies. Therefore, the addition of iron (III) oxide-hydroxide into orange peel powder in this study helped to increase the maximum adsorption capacity of orange peel material. In addition, the raw material plays the main role in lead adsorption resulting in different lead adsorption capacities. Furthermore, comparing lead adsorption by orange peels of this study and the study of Chinyelu et al. found that the material size might be another effect to lead adsorption which the smaller material size could highly remove lead. However, the costs of material synthesis and material separation after the treatment of small materials are higher than big materials, so the operation cost might be a concern for real applications.

**Table 6:** Comparison of the maximum adsorption capacity (*q*_m_) of various waste peels for lead adsorption.

Material	Modifications	Condition	*q*_m_ (mg/g)	References
Pomelo peels	–	0.5 g, 210 min, pH 2.5, 10–30 mg/L, 50 mL, 30 °C	2.14	^18^
Potato peels	–	0.3 g, 250 µm, 30 min, 50 mL, 20–40 mg/L	4.99	^16^
Orange peels	–	5 g, 0.3 µm, 45 min, 10–50 mg/L, 100 mL	5.76	^30^
Watermelon peels	–	0.25 g, 250 µm, 60–120 mg/L, 15 mL, 30 °C	10.10	^19^
Banana peels	–	2 g, 250 µm, 20 min, pH 5, 30–80 µg/L, 50 mL, 25 °C	2.18	^68^
Banana peels	–	0.5 g, 200 and 400 µm, 2–10 mg/L, 100 mL	4.64	^27^
Banana peels	Activated carbon coated with Al_2_O_3_	0.1 g, 40 min, pH 6, 5–100 mg/L, 20 mL, 25 °C	57.00	^44^
Lemon peels	–	4 g, 125 µm, 6 h, pH 7, 10–70 mg/L, 100 mL, 25 °C	1.91	^5^
Lemon peels	Iron (III) oxide-hydroxide	3 g, 125 µm, 6 h, pH 7, 10–70 mg/L, 100 mL, 25 °C	3.52	^5^
OP	–	4 g, 125 µm, 6 h, pH 5, 10–70 mg/L, 100 mL, 25 °C	1.88	This study
OPF	Iron (III) oxide-hydroxide	3 g, 125 µm, 6 h, pH 5, 10–70 mg/L, 100 mL, 25 °C	2.30	This study

### Adsorption kinetics

**Figure 9:**
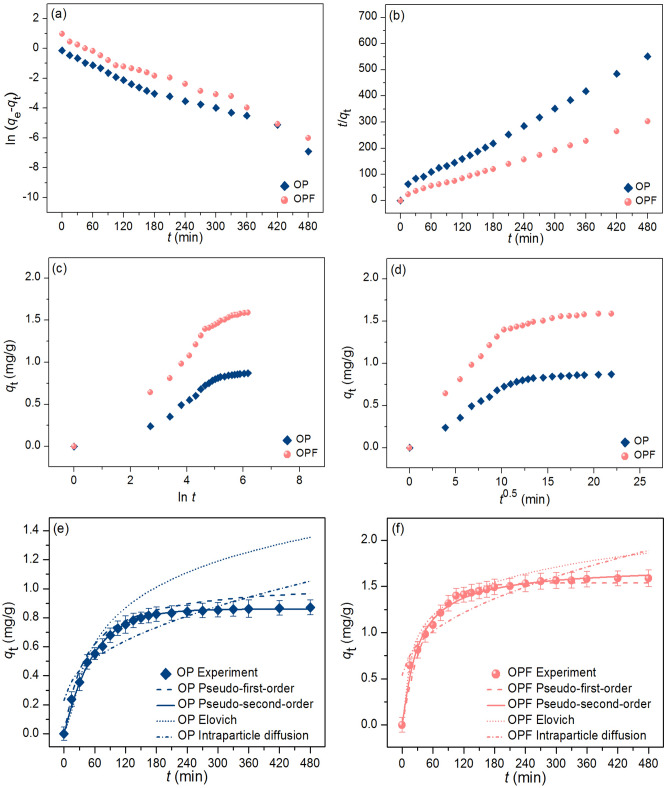
Graphs of (**a**) linear pseudo-first-order, (**b**) linear pseudo-second-order, (**c**) linear elovich model, (**d**) linear intraparticle diffusion, and (**e, f**) nonlinear kinetic models of OP and OPF for lead adsorptions.

The adsorption mechanism and reaction rate of OP and OPF for lead adsorptions were investigated by linear and nonlinear kinetic models of a pseudo-first-order kinetic model, pseudo-second-order kinetic model, elovich model, and intraparticle diffusion. For linear models, they were plotted by ln (*q*_e_ − *q*_t_) versus time (*t*), *t*/*q*_t_ versus time (*t*), *q*_t_ versus ln *t*, and *q*_t_ versus time (*t*^0.5^) for a pseudo-first-order kinetic, pseudo-second-order kinetic, elovich, and intraparticle diffusion models, respectively. For nonlinear models, they were plotted by *q*_t_ versus time (*t*). The plotting graph results are illustrated in Fig. 9a–f, and the adsorption kinetic parameters are presented in Table 7.

**Table 7:** The comparison of linear and nonlinear kinetic parameters for lead adsorptions on OP and OPF.

Regression method	Kinetic model	Parameter	OP	OPF
Linear	Pseudo-first-order	*q*_e_ (mg/g)	0.988	1.091
		*k*_1_ (min^−1^)	0.012	0.013
		*R*^2^	0.975	0.985
	Pseudo-second-order	*q*_e_ (mg/g)	1.044	1.853
		*k*_2_ (g/mg·min)	0.026	0.028
		*R*^2^	0.991	0.994
	Elovich	*α* (mg/g/min)	0.748	0.935
		*β* (g/mg)	6.053	3.560
		*R*^2^	0.941	0.968
	Intraparticle diffusion	*k*_i_ (mg/g·min^0.5^)	0.038	0.062
		*C*_i_ (mg/g)	0.230	0.540
		*R*^2^	0.803	0.772
Nonlinear	Pseudo-first-order	*q*_e_ (mg/g)	0.994	1.039
		*k*_1_ (min^−1^)	0.014	0.018
		*R*^2^	0.976	0.988
		*R*^2^_adj_	0.974	0.987
		RMSE	0.089	0.057
	Pseudo-second-order	*q*_e_ (mg/g)	1.050	1.872
		*k*_2_ (g/mg·min)	0.023	0.029
		*R*^2^	0.992	0.993
		*R*^2^_adj_	0.991	0.992
		RMSE	0.062	0.035
	Elovich	*α* (mg/g/min)	0.752	0.950
		*β* (g/mg)	6.132	3.675
		*R*^2^	0.943	0.970
		*R*^2^_adj_	0.941	0.968
		RMSE	0.252	0.130
	Intraparticle diffusion	*k*_i_ (mg/g·min^0.5^)	0.041	0.067
		*C*_i_ (mg/g)	0.232	0.553
		*R*^2^	0.807	0.774
		*R*^2^_adj_	0.805	0.772
		RMSE	0.109	0.196

For linear models, the adsorption capacities (*q*_e_) of OP and OPF on a pseudo-first-order kinetic model were 0.988 and 1.091 mg/g, and their reaction of rate constants (*k*_1_) were 0.012 and 0.013 min^−1^. For a pseudo-second-order kinetic model, the adsorption capacities (*q*_e_) of OP and OPF were 1.044 and 1.853 mg/g, and their reaction of rate constants (*k*_2_) were 0.026 and 0.028 g/mg·min. For the elovich model, the initial adsorption rates (α) of OP and OPF were 0.748 and 0.935 mg/g/min, and their extents of surface coverage (*β*) were 6.053 and 3.560 g/mg. For the intraparticle diffusion model, the reaction of rate constants (*k*_i_) of OP and OPF were 0.038 and 0.062 mg/g·min^0.5^, and their constant *C*_i_ values were 0.230 and 0.540 mg/g. *R*^2^ values of OP and OPF on pseudo-first-order and pseudo-second-order kinetic models were 0.975, 0.985 and 0.991, 0.994, respectively. In addition, *R*^2^ values of OP and OPF on elovich and intraparticle diffusion models were 0.941, 0.968 and 0.803, 0.772, respectively.

For nonlinear models, the adsorption capacities (*q*_e_) of OP and OPF on a pseudo-first-order kinetic model were 0.994 and 1.039 mg/g, and their reaction of rate constants (*k*_1_) were 0.014 and 0.018 min^−1^. For a pseudo-second-order kinetic model, the adsorption capacities (*q*_e_) of OP and OPF were 1.050 and 1.872 mg/g, and their reaction of rate constants (*k*_2_) were 0.023 and 0.029 g/mg·min. For the elovich model, the initial adsorption rates (α) of OP and OPF were 0.752 and 0.950 mg/g/min, and their extents of surface coverage (*β*) were 6.132 and 3.675 g/mg. For the intraparticle diffusion model, the reaction of rate constants (*k*_i_) of OP and OPF were 0.041 and 0.067 mg/g·min^0.5^, and their constant *C*_i_ values were 0.232 and 0.553 mg/g. *R*^2^ values of OP and OPF on pseudo-first-order and pseudo-second-order kinetic models were 0.976, 0.988 and 0.992, 0.993, respectively. In addition, *R*^2^ values of OP and OPF on elovich and intraparticle diffusion models were 0.943, 0.970 and 0.807, 0.774, respectively. Moreover, *R*^2^_adj_ of OP and OPF in nonlinear pseudo-first-order and pseudo-second-order kinetic models were 0.974, 0.987 and 0.991, 0.992, respectively. *R*^2^_adj_ of OP and OPF in nonlinear elovich and intraparticle diffusion models were 0.941, 0.968 and 0.805 0.772, respectively.

For *R*^2^ value consideration, since *R*^2^ values of OP and OPF in both linear and nonlinear pseudo-second-order kinetic models were higher than pseudo-first-order kinetic, elovich, and intraparticle diffusion models, so their adsorption rate and mechanism of both materials corresponded to pseudo-second-order kinetic model with relating to a chemisorption process with heterogeneous adsorption. Moreover, it also recommends plotting kinetic graphs in both linear and nonlinear models for confirming results and protecting against data mistranslations^69–72^.

### Desorption experiments

**Figure 10:**
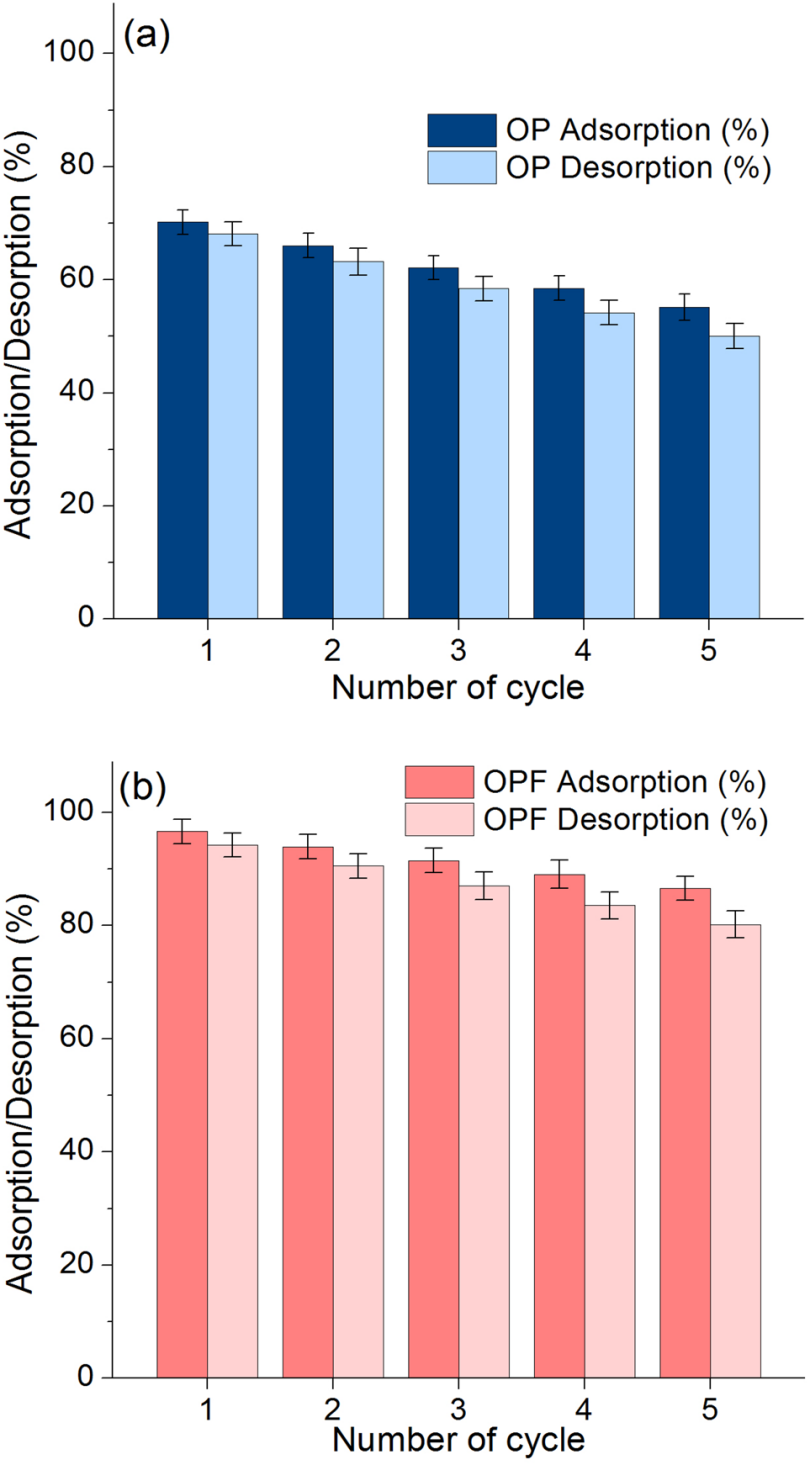
The desorption experiments of (**a**) OP and (**b**) OPF.

The possible reuses of OP and OPF are important points to estimate the cost and economic feasibility of industrial applications which were studied through the desorption experiments. The lead adsorption–desorption in 5 cycles is designed to investigate their reusable abilities, and their results are illustrated in Fig. 10a,b. OP could be reused in 5 cycles with high adsorption and desorption in ranges of 55.14–70.17% and 50.06–68.09%, respectively which adsorption and desorption were decreased by approximately 15% and 18%, respectively shown in Fig. 10a. OPF also confirmed to be reusability in 5 cycles with high adsorption and desorption in ranges of 86.57–96.64% and 80.20–94.23%, respectively which adsorption and desorption were decreased by approximately 10% and 14%, respectively shown in Fig. 10b. Therefore, both materials are potential materials for lead adsorption with the reusability of more than 5 cycles by more than 55%, and they can be further applied to industrial applications.

## The possible mechanisms of lead adsorptions by OP and OPF

**Figure 11:**
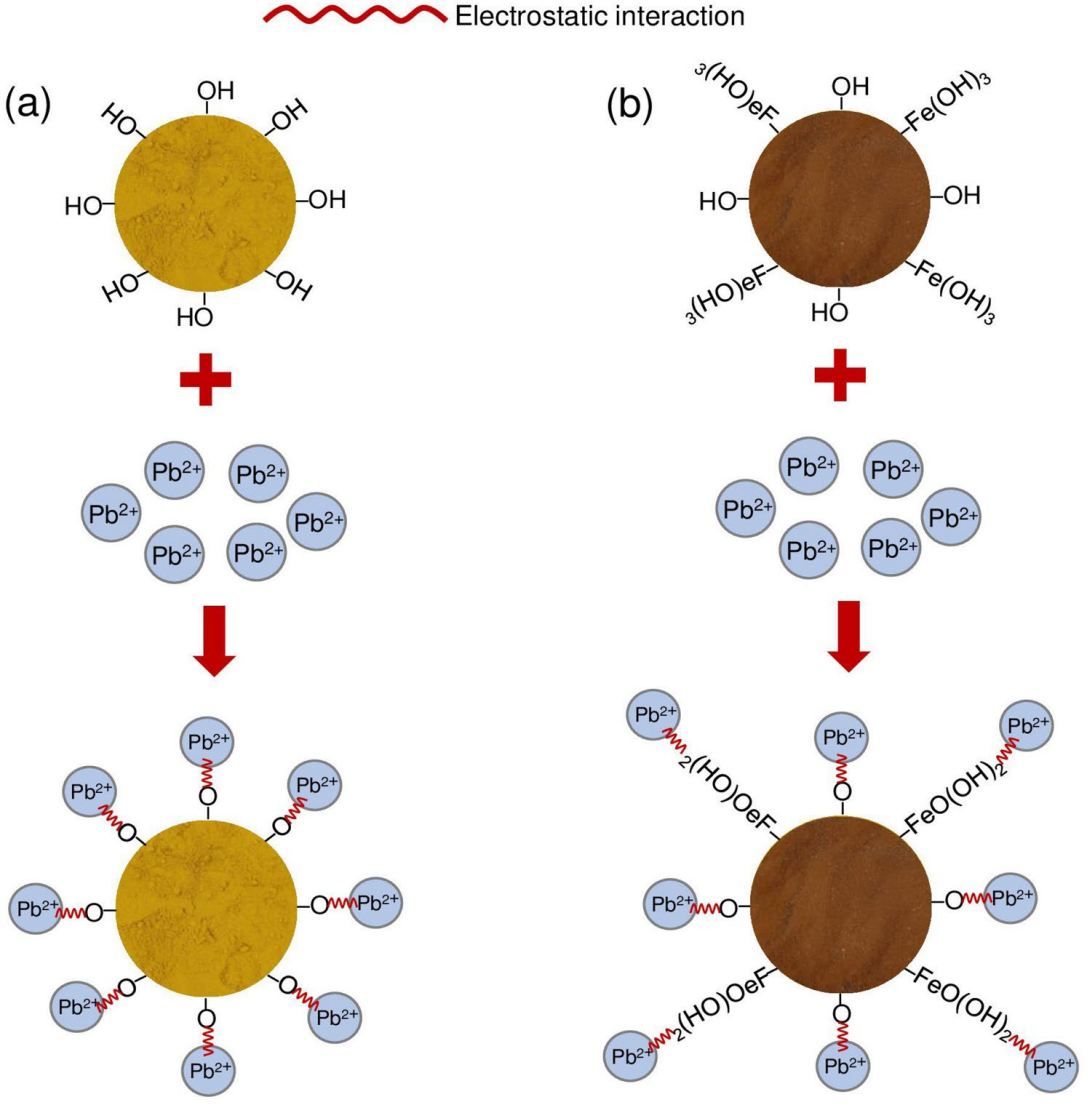
Possible mechanism of lead adsorptions on (**a**) OP and (**b**) OPF.

The possible mechanisms of lead adsorptions on OP and OPF are demonstrated in Fig. 11a,b which are modified an idea from the previous studies^6,9,10^. The main structures of OP and OPF are composed of cellulose, hemicellulose, pectin, and lignin including the main functional groups of the hydroxyl group (–OH). Since iron (III) oxide-hydroxide was added into OP to be OPF, the complex compound of OP∙Fe(OH)_3_ was found on the surface from adding iron (III) oxide-hydroxide into OP by sharing electrons with –OH of OP. The possible mechanism of lead adsorptions by OP and OPF might occur from donating a proton (H^+^) from –OH or OP∙Fe(OH)_3_ of the main chemical compounds for capturing lead (II) ions (Pb^2+^) instead of H^+^ from a process of electrostatic interaction^6^.

## Conclusion

Orange peel powder (OP) and orange peel powder doped iron (III) oxide-hydroxide (OPF) were successfully synthesized for lead adsorption in an aqueous solution. The specific surface area and pore volume of OPF were higher than OP, whereas its pore size was smaller than OP. They were semi-crystalline structures that presented the specific cellulose peaks, and OPF also detected the specific iron (III) oxide-hydroxide peaks. The surface morphologies of OP and OPF were irregular and porous surfaces. Three main chemical compositions of OP and OPF were carbon (C), oxygen (O), and calcium (Ca), whereas iron (Fe), sodium (Na), and chloride (Cl) were only detected in OPF with adding iron (III) oxide-hydroxide. Six main chemical functional groups of O–H, C–H, C=C, C–O, C=O, and –COOH were detected in both materials whereas Fe–O was only found in OPF. The pH_pzc_ of OP and OPF were 3.74 and 4.46. For batch experiments, the optimum conditions of OP and OPF were 4 g, 6 h, pH 5, 50 mg/L and 3 g, 6 h, pH 5, 50 mg/L, and their lead removal efficiencies were 69.78% and 95.29%. As a result, OPF demonstrated a higher lead removal efficiency than OP because it spent less material dosage and gave a high percentage of lead removal than OP. Therefore, adding iron (III) oxide-hydroxide helped to improve orange peel efficiency for lead adsorption. For the isotherm study, both materials corresponded to the Freundlich model correlated to a physicochemical process. For the kinetic study, they corresponded to a pseudo-second-order kinetic model related to a chemisorption process with heterogeneous adsorption. Moreover, both materials could be reusable for more than 5 cycles for lead adsorptions of more than 55%. Therefore, OP and OPF were high-potential materials for lead adsorptions in an aqueous solution, and OPF demonstrated the highest lead removal efficiency. Therefore, OPF was suitable to apply for industrial wastewater treatment applications in the future.

In future works, the continuous flow study also needs to study for further industrial applications, and the competing ions such as sodium (Na^+^), magnesium (Mg^2+^), and natural organic matter (NOM) contaminated in real wastewater should be investigated to confirm the specific lead adsorption by OP or OPF.

## Data Availability

The datasets used and/or analyzed during the current study are available from the corresponding author upon reasonable request.
